# Outcomes of ureteroscopy and internal ureteral stent for pregnancy with urolithiasis: a systematic review and meta-analysis

**DOI:** 10.1186/s12894-022-01100-w

**Published:** 2022-09-14

**Authors:** Xingwei Jin, Boke Liu, Yunqi Xiong, Yuanchun Wang, Weichao Tu, Yuan Shao, Lin Zhang, Dawei Wang

**Affiliations:** 1 Department of Urology, School of Medicine, Ruijin Hospital, Shanghai Jiaotong University, No. 197 Ruijin Er Road, Shanghai, 200025 China; 2 Department of Gynaecology and Obstetrics, Shanghai TCM College Affiliated Shu Guang Hospital, Shanghai, China; 3 Monash Health, Monash Medical Centre, Clayton, VIC 3168 Australia; 4 School of Population Medicine and Public Health, Chinese Academy of Medical Science & Peking Union Medical College, Beijing, 100730 China; 5 Centre for Epidemiology and Biostatistics, Melbourne School of Population and Global Health, The University of Melbourne, Parkville, VIC 3100 Australia; 6 Victorian Comprehensive Cancer Centre, The University of Melbourne Centre for Cancer Research, Parkville, VIC 3100 Australia

**Keywords:** Pregnancy, Urolithiasis, Double-J stent, Ureteroscopy

## Abstract

**Objectives:**

To investigate the outcomes of internal ureteral stents in comparison with ureteroscopy (URS) for pregnant women with urolithiasis.

**Data sources:**

Relevant studies published from January 1980 to June 2022 were identified through systematic literature searches of MEDLINE, EMBASE, Web of Science and the Cochrane Library.

**Methods of study selection:**

A total of 499 studies were initially identified. We included pregnant women in any stages of gestation who underwent double-J (D-J) stent insertion only or ureteroscopy for the treatment of urolithiasis; for a study to be included, the number of participants needed to exceed 10. This systematic review was registered on the PROSPERO website (Reference: CRD42020195607).

**Results:**

A total of 25 studies were identified with 131 cases undergoing serial stenting and 789 cases undergoing URS. The pooled operative success rate was 97% for D-J stent insertion and 99% for URS. Only a few patients passed stones spontaneously after serial D-J stenting. The pooled stone free rate (SFR) in URS operations was about 91%. For internal ureteral stent therapy, the rate of normal fertility outcomes was 99%, although the pooled incidence of complications was approximately 45%. For group receiving URS treatment, the rate of normal fertility outcome was 99% and the pooled incidence of complications was approximately 1%. However, the pooled rate of premature birth and abortion were the similar between the two groups (< 1%); the rate of serious complications was also similar between the two groups.

**Conclusions:**

Although internal ureteral stents may cause more minor complications, both ureteroscopy and internal ureteral stents showed had low rates of adverse effects on fertility outcomes when used to treat pregnant women with symptomatic urolithiasis. Evidence suggests that URS may have a greater advantage for pregnant patients with urinary stones when conditions permit. Since, it has been proven to be safe and effective, internal ureteral stents could be considered in emergency or other special situations.

**Supplementary Information:**

The online version contains supplementary material available at 10.1186/s12894-022-01100-w.

## Introduction

The incidence of pregnant women with symptomatic urinary tract stones is reported to range from 1 in 2000 to 1 in 200 [1]. Symptomatic urolithiasis can lead to renal colic, urinary tract infection and ureteral obstruction, thus, creating significant morbidity and potential mortality for both the mother and the fetus. The main complications are pre-term delivery and premature rupture of the membranes; this can create serious health risks for the fetus [2, 3]. It is important for urologists and obstetricians to be aware of how to manage this condition.

When managing a pregnant patient with urolithiasis, conservative management is favoured where possible. Surgical intervention is available for those that do not improve with conservative measures [4]. Ureteroscopy (URS) and internal ureteral stents are the most widely used treatments for pregnant females with symptomatic urolithiasis [5]. The insertion of a double-J (D-J) stent until definitive treatment during the postpartum period is a temporary measure and studies relating to this procedure are scarce. With continuous advancement in endoscopic technology and endourological techniques, URS has become the first-line treatment for the management of ureteric stones in pregnancy. Although the latest 2020 European Association of Urology (EAU) guidelines recommends URS as a reasonable alternative option [6], there is still a lack of evidential evaluation for URS in comparison with internal ureteral stents. In this systematic review and meta-analysis, we provide an up-to-date comparison between the outcomes of internal ureteral stent and URS treatments for pregnant women with urolithiasis.

## Methods

We performed a systematic review according to a pre-determined protocol which was reported in accordance with the Preferred Reporting Items for Systematic Reviews and Meta-Analyses Protocols (PRISMA-P) guidelines [7]. We registered our systematic review on the PROSPERO website (www.york.ac.uk/inst/crd, registration number: CRD42020195607). Two reviewers independently undertook the literature search (XJ and BL), assessment for eligibility (XJ and BL), data extraction (YS and WT) and qualitative assessment (DW and YX). Any inconsistencies between the two reviewers were reviewed by a third reviewer (LZ) and resolved by consensus. If data sources were duplicated in more than one study, only the original study was included in the meta-analysis as per consensus among all three reviewers (XJ, BL and LZ).

### The definition of PICOS used in this study

Participants: Pregnant women of any gestation with urolithiasis.

Intervention: D-J stent insertion only.

Comparators (controls): URS operation for lithotripsy/stone extraction/exploration.

Outcome: Fertility results and complications.

Study design: RCTs and observational studies (case–control, cross-sectional and cohort) were included in this systematic review and meta-analysis.

### Eligibility criteria

Studies were included if they (1) Featured pregnant women in any stage of pregnancy and underwent D-J stent insertion only or ureteroscopy for the treatment of urolithiasis, (2) Had been published between January 1980 and June 2022, and (3) Featured more than 10 participants.

Studies were excluded if they (1) Were reviews, comments, letters, guidelines, or meta-analyses (2) Lacked data relating to pregnancy or interventions, (3) lacked photography, equipment evaluation or diagnosis criteria for urolithiasis in pregnancy, (4) Involved research on neonates, (5) Involved physiological hydronephrosis without stone disease, and (6) If they featured extracorporeal shock wave lithotripsy, percutaneous nephrostomy or other treatments for pregnancy with urolithiasis.

### Search strategy

We conducted a literature search using PubMed (MEDLINE), Embase, Web of Science and the Cochrane Library of articles published from January 1980 to June 2022. Medical Subject Heading (MeSH) terms were used in conjunction with the following keywords: (Pregnanc* or Pregnancy or Pregnant or Gestation* or Pregnant woman or Mother*) *AND* (Urinary Calcul* OR Urinary Calculi OR Urinary Calculus OR Urinary Stone* OR Urinary Tract Stone* OR Ureteral Calcul* OR Ureteral Calculi OR Ureteral Calculus OR Kidney Calcul* OR Kidney Calculi OR Kidney Calculus OR Nephrolith OR Renal Calcul* OR Renal Calculi OR Renal Calculus OR Kidney Stone* OR Staghorn Calcul* OR Staghorn Calculi OR Staghorn Calculus OR Urinary Lithiasis) *AND* (Ureteroscopies OR Ureteroscopic OR Ureteroscopic Surgical OR Ureteroscopic Surgical Procedure* OR Ureteroscopic Surgery OR Ureteroscopy) *AND* (Double-J stent OR Ureteral stent OR Ureteral double-J stent OR Ureteral D-J stent OR Double J ureteral stent OR D-J ureteral stent OR stent OR D-J stent). Full search strings are presented in Additional file 1: Table S1. References from relevant articles, editorials, conference abstracts, letters, and reviews were thoroughly reviewed to identify additional studies. Full manuscripts of every article with a relevant title and abstract were then reviewed for eligibility.

### Data extraction and qualitative assessment

Two reviewers (YS, WT) independently extracted the following study-level characteristics from each eligible study: first author, year of publication, country where the study was conducted, journal, study period, age, trimester, diagnose method, stone location and size, anaesthetic method, intervention and sample size, operation success rate, stone free rate (SFR), fertility outcome, complications and follow-up pattern. Two groups were set as different treatment procedures: an internal ureteral stent (D-J stent) therapy group and a URS group. Fertility outcomes included normal delivery, cesarean section, premature labor, abortion and others (which are listed in the tables below). Final fertility results were used to assess treatments, and only premature labor and abortion were considered as serious fertility outcomes (which imply failure to save the fetus). Fertility outcomes and complications were also assessed with the Clavien-Dindo classification, as shown in Additional file 1: Table S2. A Clavien-Dindo classification of III-V was regarded as a serious complication.

We applied the Newcastle–Ottawa Scale (NOS) quality assessment tool to evaluate the quality of the selected observational studies. This tool was used to measure key aspects of the methodology in selected studies with regards to design quality and the risk of biased estimates based on three design criteria: (1) Selection of study participants, (2) Comparability of study groups, and (3) The assessment of outcome and exposure with a star system (with a maximum of 9 stars). We judged studies that received a score of 7–9 stars to be of a low risk of bias, studies that scored 4–6 stars to be of a medium risk, and those that scored 3 or less to be of a high risk of bias. A funnel plot was used to assess publication bias. Any disagreement on the data extraction and quality assessment of the studies were resolved through comprehensive discussion (DW, YX and LZ).

### Statistical analysis

Study-specific prevalence rate estimates were combined using a random-effects model that considered within-study and between-study variations. Corresponding 95% confidence intervals (CIs) were extracted directly from articles where available. Statistical heterogeneity among studies was evaluated using Cochran’s Q test and the *I*^*2*^ statistic, with values of 25%, 50%, and 75% representing low, moderate and high heterogeneity, respectively. The criterion for identifying heterogeneity was *P* < 0.05 for the Q test.

An estimation of publication bias was evaluated by Begg’s funnel plot, in which the standard error (SE) of the log odds ratio (OR) of each study was plotted against its log OR. An asymmetrical plot suggested potential publication bias. Egger's linear regression test was used to evaluate funnel plot asymmetry on the natural logarithm scale of the rates. All statistical analyses were performed using Stata (version 14.2; StataCorp LP, College Station, Texas). All *P* values were two-sided, and *P* < 0.05 was considered as statistically significant.

## Results

### Selection of studies

**Fig. 1:**
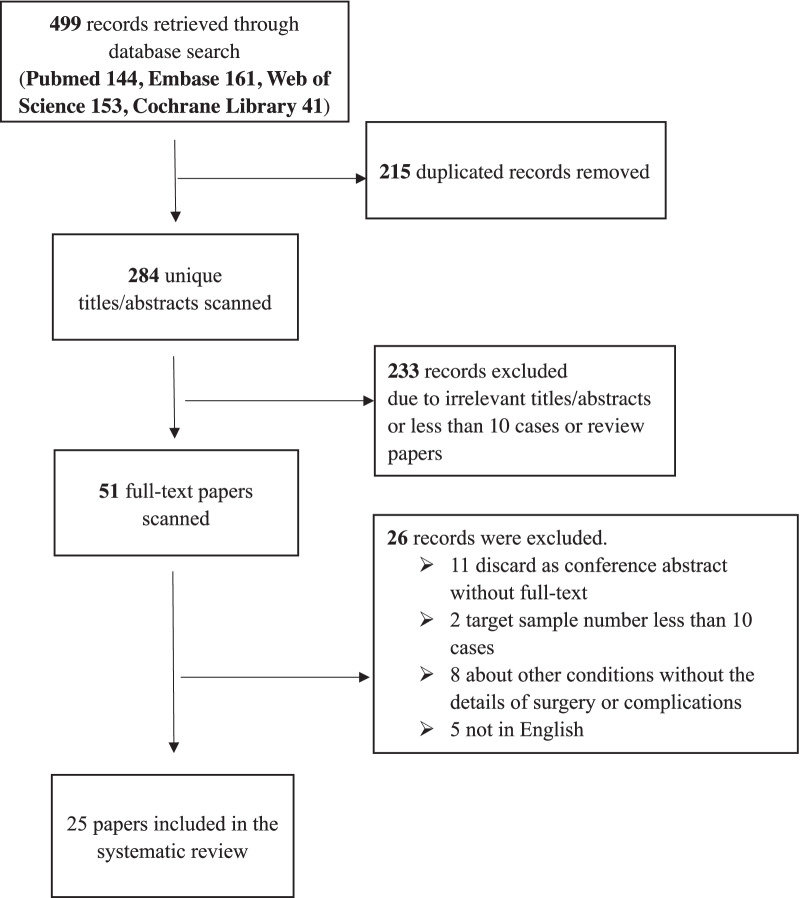
PRISMA flow diagram of study selection for meta-analysis

A detailed PRISMA flow diagram showing the literature search and inclusion criteria is given in Fig. 1. A total of 499 studies were initially identified with this literature search (144 from PubMed, 161 from Embase, 153 from Web of Science and 41 from Cochrane Library). Of these, 215 studies were excluded due to duplication and 233 were excluded after screening the titles and abstracts. Then, 26 other studies were excluded after full-text review. Finally, a total of 25 studies were identified as eligible for systematic review and meta-analysis.

The time span of the 25 studies included in this analysis was 1995–2018, and the research period of cases ranged from 1984 to 2016. Common information from publications is shown in Table 1. Of the 25 studies, one was from Norway [8], one from Italy [9], two from America [10], one from Brazil [11], one from Pakistan [12], four from Egypt [13, 20, 27, 29], five from China [14, 22, 28, 30, 32], six from Turkey [15–18, 21, 25], two from Iran [23, 31], one from Iraq [24] and one from Romania [26]. The age range of the patients involved was 16 to 41 years and urolithiasis occurred most often in the second trimester. Ultrasound was the most commonly used diagnostic method. The most common sites for calculi were the distal ureter, medium ureter and proximal ureter. The mean stone size was between 6 and 17 mm.

**Table 1:** Summary of characteristic for studies included in the meta-analysis

First author	Year	Country, Continent	Journal	Period	Age range	Trimester	Diagnosis method	Stone location (No.)	Stone size, mm (mean/SD,range)
Ulvik[8]	1995	Norway, Europe	Journal of Urology	September 1984-December 1994	27 (20–41)	4–14 weeks in 3; 15–28 weeks in 9; 29–37 weeks in 12	KUB 1 positive in 6; US 3 positive in 21 (hydronephrosis 21 in 21)	Not mentioned	Not mentioned
Scarpa[9]	1996	Italy, Europe	Journal of Urology	3-years period	24 (16–30)	20–34	US\symptoms\urinalysis	Not mentioned	Not mentioned
Parulkar[10]	1998	America, North America	Journal of Urology	January 1984-November 1995	27 (< 18y 2; 18-20y 4; 20-30y 43; 30-40y 21)	First trimester in 3; second trimester in 23; third trimester in 44	US 40 positive in 65; IVP 5 positive in 5	Not mentioned	US 0.7 (0.4–1.6); IVP 0.55 (0.4–0.7)
Lemos[11]	2002	Brazil, South America	International Braz J Urol	Not mentioned	28 (20–34)	18 (12–34)	US 12 positive in 12; ureteroscopy 13 positive in 14	Proximal ureter in 1; medium ureter in 4; distal ureter in 12; 1 missed	6 (4–12)
Rana[12]	2009	Pakistan, Asia	Urology	1997—2007	22 (18–27)	20 (14–34) First trimester in 1; second trimester in 11; third trimester in 7	US in 11; KUB in 1	Proximal ureter in 11; distal ureter in 8;	11 (8–18)
Elgamasy[13]	2010	Egypt, Africa	BJU International	June 2003- June 2008	25.9 (18–38)	25.9 (24–30)	US 12 positive in 15; RU 14 positive in 15,	Proximal ureter in 2; medium ureter in 2; distal ureter in 10;	Not mentioned
Liu[14]	2011	China, Asia	Journal of Huazhong University of Science and Technology-Medical Sciences	January 2004—December 2009	26.7 (18–37)	23.45 (4–38)	US in 24	6 bilateral; 8 left; 10 right (surgery group)	Not mentioned
Polat[15]	2011	Turkey, Asia	Urological Research	2007–2009	25 (19–34)	30 (23–35) second trimester in 8; third trimester in 8	US in 11	Proximal ureter in 5; distal ureter in 6;	9.45 (5–12)
Atar[16]	2012	Turkey, Asia	International Journal of Surgery	December 2010-July 2011	26 (19–40)	24 (16–33)	US for 8, ureteroscopy for all	Proximal ureter in 5; medium ureter in 5; distal ureter in 7; no stone in 2	8 (5–19)
Bozkurt[17]	2012	Turkey, Asia	Urological Research	April 2005-Nocember 2010	27.8 (20–39)	24 (15–34)	US 16 positive; all 32 positive underwent URS	Proximal ureter in 8; medium ureter in 9; distal ureter in 10; no stone in 5	8 (5–19, in 16 US positive cases)
Hoscan[18]	2012	Turkey, Asia	Urology	2001–2011	24 (17–37)	26 (12–38)	URS 34 positive in 57	Proximal ureter in 8; medium ureter in 6; distal ureter in 20	7 (4–13)
Johnson[19]	2012	America, North America	Journal of Urology	Not mentioned	27	24.7	Low dose CT in 23; US in 18; MRI in 5	Not mentioned	7.8 (3–25)
Abdel[20]	2013	Egypt, Africa	Urology Annals	April 2008-March 2011	23 (19–28)	25 (16–35)	Clinical presentation and US; MRI in 3	Proximal ureter in 2; medium ureter in 5; distal ureter in 10	17 (12–21)
Bozkurt[21]	2013	Turkey, Asia	Urolithiasis	April 2005-Setemper 2011	27.41 ± 5.79	23.2 ± 4.6 (13–34)	Clinical presentation, presence of microscopic hematuria in urinalysis and US	Proximal ureter in 13; medium ureter in 13; distal ureter in 15	9.78 ± 3.47
Song[22]	2013	China, Asia	International Journal of Gynecology and Obstetrics	April 2001—July 2012	27.2§	26.5§	US 23 positive in 54; MRI 25 positive in 31	Proximal ureter in 10; distal ureter in 44	13.14 (7–22)
					27.1¶	26.3¶			
Keshvari[23]	2013	Iran, Asia	Nephro-Urology Monthly	June 2003-April 2011	23 ± 2 (19–34)	24 ± 3 (12–36) First trimester in 2; second trimester in 26; third trimester in 16	US in 44; IVP in 2	Proximal ureter in 2; medium ureter in 10; distal ureter in 36	Not mentioned
Ngai[24]	2013	Iraq, Asia	Arab Journal of Urology	March 2008-March 2010	27.2 (18–38)	First trimester in 5; second trimester in 15; third trimester in 10	US showed hydronephrosis in 30, stone in 12	Not mentioned	Not mentioned
Adanur[25]	2014	Turkey, Asia	Archivio Italiano di Urologia e Andrologia	January 2005-December 2012	25.4 (18–41)	24.8(7–33)	US in 6; ureteroscopy for all	Proximal ureter in 6; medium ureter in 5; distal ureter in 8	9.2 (6–13) in 6 with US
Georgescu[26]	2014	Romania, Europe	Chirurgia	January 2006-January 2012	27.2 (20–37)	First trimester in 6; second trimester in 32; third trimester in 16	US stone 18 positive in 54	Proximal ureter in 11; medium ureter in 8; distal ureter in 14	8 (4–16)
								Not mentioned	
Teleb[27]	2014	Egypt, Africa	Arab Journal of Urology	October 2006-December 2013	26.6 (SD 4.65)§	24.1 (SD 5.44)§	US 31 positive in 43	Middle ureter in 9§; distal ureter in 13§	Not mentioned
					25.5 (SD 4.26)¶	25.7 (SD 4.95)¶		Middle ureter in 7¶; distal ureter in 14¶	
Wang[28]	2014	China, Asia	Urology	February 2006-Setemper 2012	26 (17–39)	29(17–39) First trimester in 2; second trimester in 36; third trimester in 49	US in 79, MRI in 8,	Left side in 48, Right side in 39	8 (5–19)
Fathelbab[29]	2016	Egypt, Africa	African Journal of Urology	April 2006-October 2013	23 (19–37)	First trimester in 4; second trimester in 23; third trimester in 14	Diagnostic ureteroscopy 36 positive in 41	Proximal ureter in 7; distal ureter in 29	8.9 (5–16)
Zhang[30]	2016	China, Asia	PLoS ONE	March 2009-Setember 2014	25.5 ± 4.6 (16–41)	9–36	US and diagnostic ureteroscopy positive in 86 (only ureteroscopy in 24), negative in 31	Not mentioned	8.2 ± 0.6
Abedi[31]	2017	Iran, Asia	Journal of Lasers in Medical Sciences	January 2007-June 2016	29.3	27.3 (13–31) First trimester in 9; second trimester in 24; third trimester in 12	Clinical manifestations, urinalysis and US	Poximal ureter in 5; distal ureter in 40	7.84 (5-9 mm)
Tan[32]	2018	China, Asia	European Journal of Obstetrics and Gynecology and Reproductive Biology	January 2005-June 2015	26.7 ± 8.9§	27.5 ± 11.2§	US	Proximal ureter in 10; medium ureter in 12; distal ureter in 31	Not mentioned
					27.4 ± 10.2¶	25.9 ± 9.7¶			

^§^ means received internal ureteral stent only; ¶ means received ureteroscopy operation

### Subgroup analysis and meta-analysis

Only two studies involved D-J stent insertion only [10, 24]; 19 studies involved URS operations [8, 9, 11–21, 23, 25, 26, 29–31], and four involved both procedures [22, 27, 28, 32]. A total of 131 cases involved internal ureteral stents only and 789 cases underwent URS operations. Common results are shown in tables and occurrence rates (ORs) were calculated and compared by meta-analysis.

**Fig. 2:**
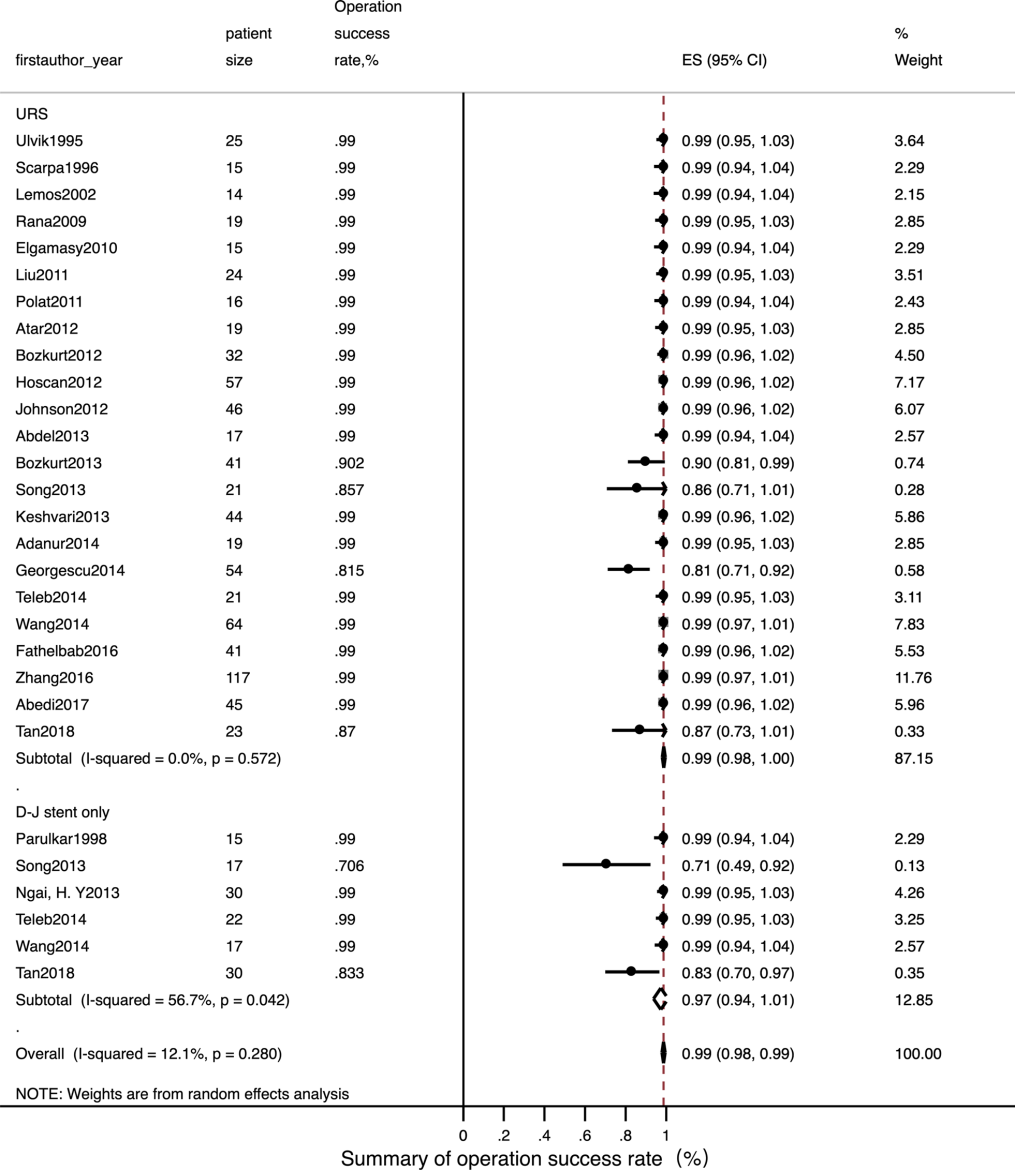
Meta-analysis about operation success rate in D-J stent therapy group and URS group

**Fig. 3:**
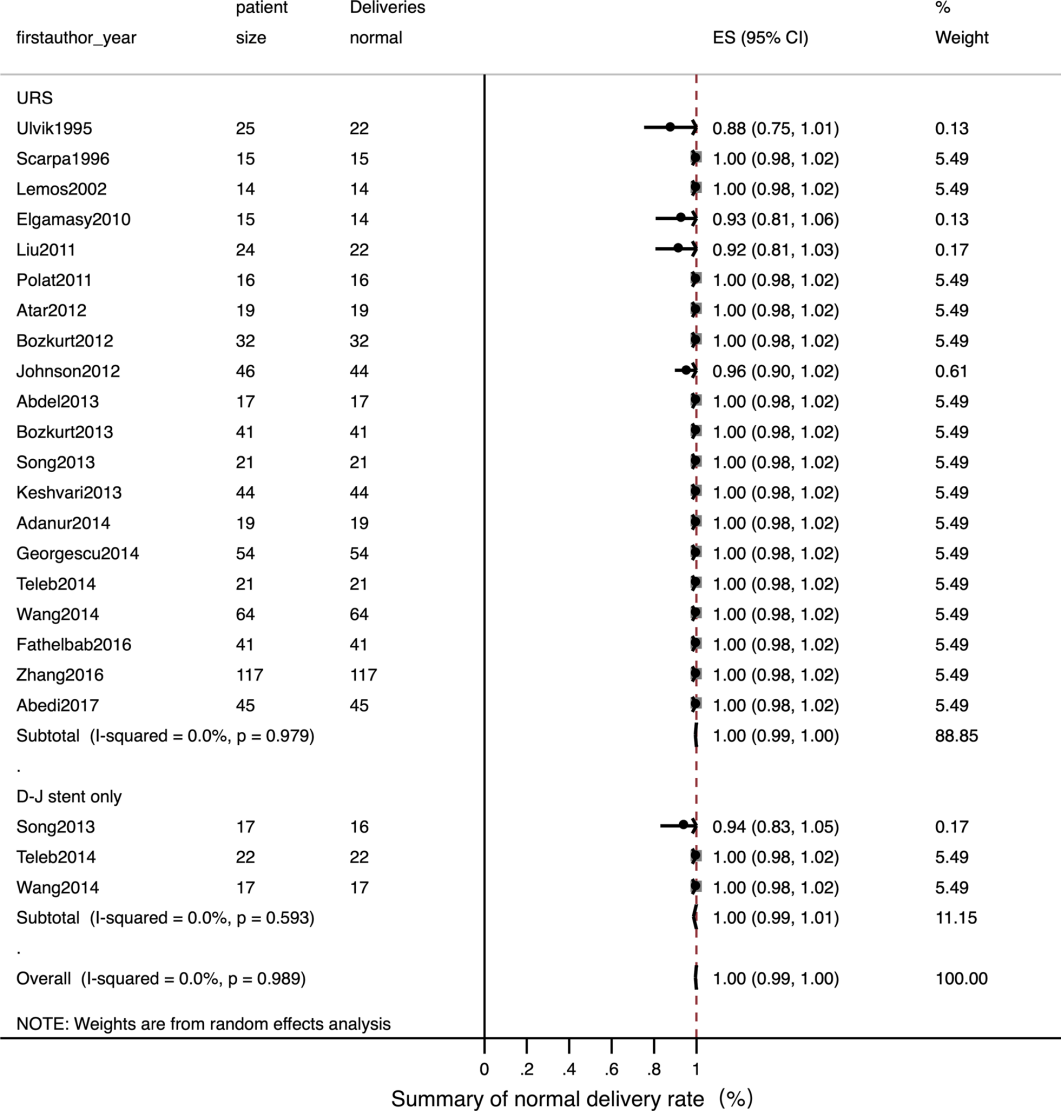
Meta-analysis about normal fertility outcome in D-J stent therapy group and URS group

**Fig. 4:**
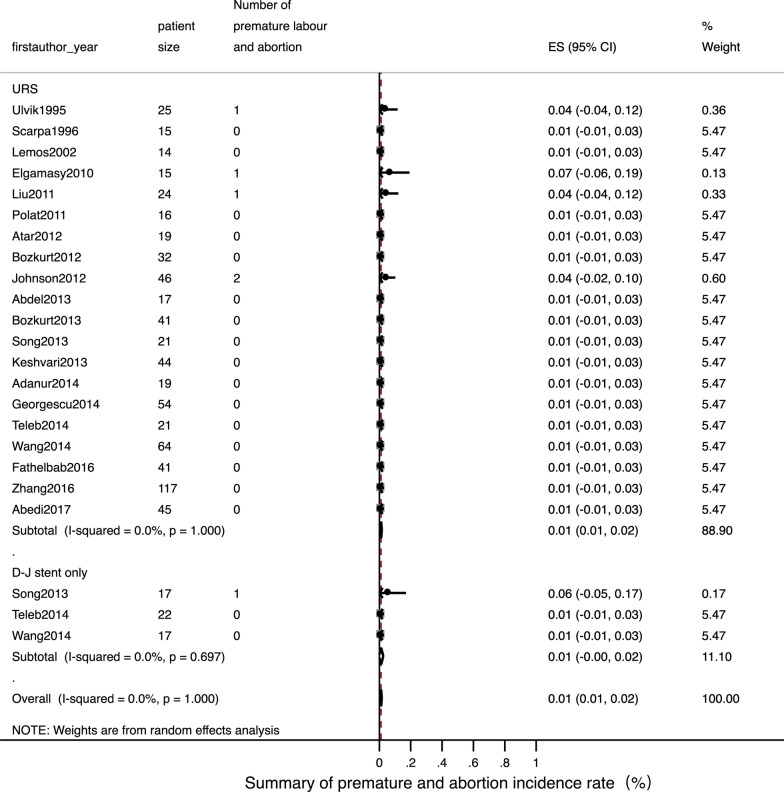
Meta-analysis about adverse pregnant outcome (premature and abortion) in D-J stent therapy group and URS group

**Fig. 5:**
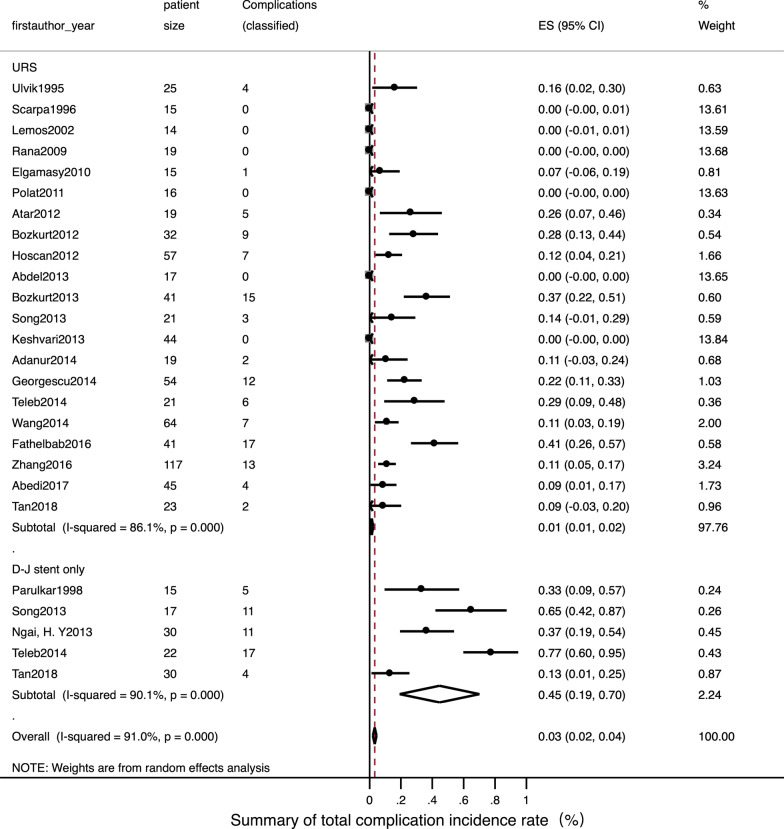
Meta-analysis about overall complications in D-J stent therapy group and URS group

**Fig. 6:**
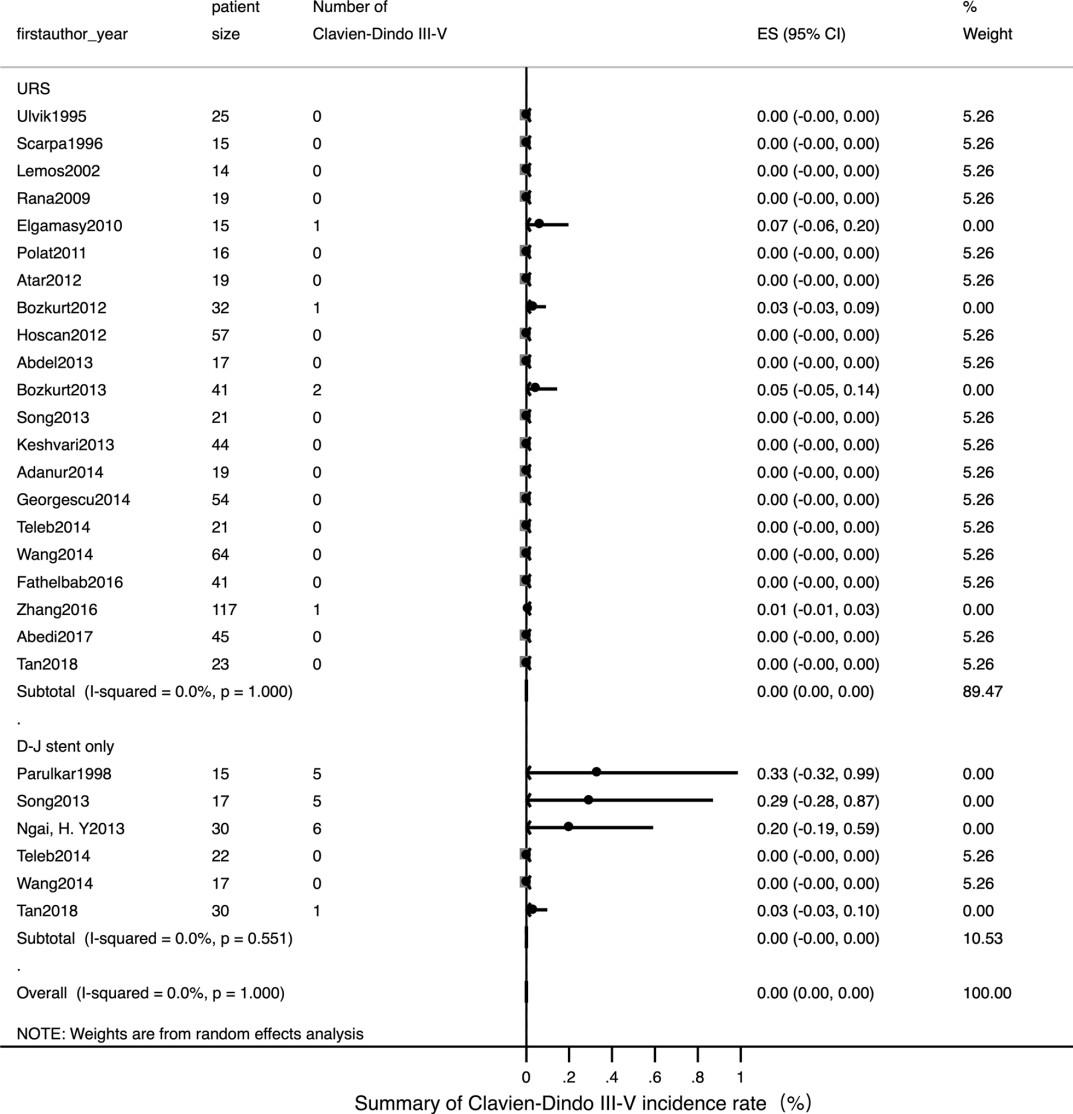
Meta-analysis about Clavien-Dindo III-V complications in D-J stent therapy group and URS group

Detailed data of internal ureteral stent therapy was showed in Table 2. The most commonly used form of anaesthesia was local. The pooled operation success rate was 97% [Fig. 2; 95% CI: 0.94–1.01]. Only one related study [22] mentioned a stone passing spontaneously in three patients; this was reported as an accident situation. The pooled ORs for a normal fertility outcome was 99% [Fig. 3; 95% CI: 0.99–1.01] and the pooled Ors for an adverse pregnant outcome (premature and abortion) was < 1% [Fig. 4; 95% CI: 0–0.02]. The pooled Ors for overall complications was 45% [Fig. 5; 95% CI: 0.19–0.70] although the pooled Ors for serious complications (Clavien-Dindo III-V) was < 1% [Fig. 6; 95% CI: 0–0].

**Table 2:** Summary of details for D-J stent therapy group

First author	Year	Anesthetic method	No. of operations (success rate)	SFR, %	Fertility outcome	Complications	Complications (classified)	Follow-up pattern
Parulkar[10]	1998	Local anesthesia	15 (100%)	\	Not mentioned	Stent slipping into bladder in 1, then repeaced; 5F stent blocked in 2,then replace to 7F; softer stent was needed in 1; calcified stent in 1	Clavien-Dindo III in 5	Not mentioned
Song[22]	2013	Local anaesthesia with lidocaine gel	17, 12 success (70.6%)	25 (3 passed stone spontaneously of 12)	16 delivered at term; preterm labor in 1	Stent-induced bladder irritation in 6, retained; encrusted stent problem in 4; passed a double-J stent in 1	Clavien-Dindo I in 6; Clavien-Dindo III in 5;	Not mentioned
Ngai[24]	2013	Local anaesthesia	30 (100%)	\	Not mentioned	Stent encrustation in 3; stent migration in 3; stent-related bladder irritation in 3; gross hematuria in 2	Clavien-Dindo I in 5; Clavien-Dindo III in 6	Renal function tests and US was arranged weekly in the first month, then monthly throughout pregnancy
Teleb[27]	2014	Spinal anaesthesia in 18, topical lidocaine anaesthesia with sedo-analgesia in 4	22 (100%)	\	All 22 delivered at term	Urinary tract infection in 4; irritative LUTS in 13	Clavien-Dindo I in 13; Clavien-Dindo II in 4	US and urinalysis every 4 weeks
Wang[28]	2014	Epidural anesthesia	17 (100%)	\	All 17 delivered at term	Urinary tract infection in 4; stent-related bladder irritation in 12; hemauria in 7	Clavien-Dindo I in 19; Clavien-Dindo II in 4	Obstetric care; clinical assessment, ultrasound examination and urine culture
Tan[32]	2018	Local anesthesia	30, 25 success (83.3%)	\	Not mentioned	Bladder irritation in 2; D-J stent drop in 1; hard removal of D-J stent in 1	Clavien-Dindo I in 3; Clavien-Dindo III in 1	Not mentioned

*SFR* stone-free rate

Detailed data relating to URS therapy is shown in Table 3. General anaesthesia and spinal anaesthesia was widely used. The pooled operation success rate was 99% [Fig. 2; 95% CI: 0.98–1]. The pooled SFR was 91% [95% CI: 0.88–0.95]. The pooled Ors for a normal fertility outcome was 99% [Fig. 3; 95% CI: 0.99–1] while the pooled Ors for an adverse pregnant outcome was < 1% [Fig. 4; 95% CI: 0.01–0.02]. The pooled Ors for overall complications was < 1% [Fig. 5; 95% CI: 0.01–0.02] and the pooled Ors for serious complications (Clavien-Dindo III-V) was < 1% [Fig. 6; 95% CI: 0–0].

**Table 3:** Summary of details for URS group

First author	Year	Anesthetic method	No. of operations (success rate)	Tool	SFR, %	Fertility outcome	Complications	Complications (classified)	Follow-up pattern
Ulvik[8]	1995	Epidural anesthesia in 23; spinal anesthesia in 1; pethidine intravenously in 1	25 (100%)	11.5F rigid URS in 23 and 9.5F rigid URS in 2	Not mentioned	Deliveries normal in 19; cesarean section in 2; seven weeks premature in 1; elective termination unrelated to ureteroscopy in 1; 1 unknown	Fever in 3 (treated with antibiotics); irritative bladder symptom in 1	Clavien-Dindo I in 1; Clavien-Dindo II in 3	IVP or ultrasound 3 months after delivery
Scarpa[9]	1996	Without anesthetic in 5; neuroleptic analgesia in 10	15 (100%)	7F rigid URS in 14 and 9.5F rigid URS in 1 (pulsed dye laser in 3, YAG laser in 3, ballistic lithotriptor in 2)	Not mentioned	All 15 delivered at term	0	0	Not mentioned
Lemos[11]	2002	Epidural anesthesia	14 (100%)	7F or 10F URS in 14 (11 removed stone with basket; 2 underwent ultrasonic lithotriptor)	100	All 14 delivered at term	0	0	Not mentioned
Rana[12]	2009	General anesthesia	19 (100%)	6.9F/8F semi-rigid URS with pneumatic lithoclast (5 need ureteral balloon dilator)	79	Not mentioned	0	0	Clinical assessment, ultrasound examination, and urine samples for culture and sensitivity
Elgamasy[13]	2010	General anaesthesia in 10; spinal anaesthesia in 5	15 (100%)	9.5F URS (5 need balloon dilation; 12 Dormia basket or pneumatic lithotripter; 2 forceps; 1 no stone)	Not mentioned	14 delivered at term; 1 premature labour (36 week)	D-J stent migration in 1	Clavien-Dindo III in 1	Patients were followed closely until delivery
Liu[14]	2011	Not mentioned	24 (100%)	Not mentioned	Not mentioned	21 natural delivery; 1 abortion; 1 cesarean	Not mentioned	Not mentioned	Not mentioned
Polat[15]	2011	General anesthesia	16 (100%), 11 with complete fragmentation of the calculi; 5 with stone push-back	9.5F semi-rigid URS with lithoclast	72.73	All 16 delivered at term	0	0	Obstetric care; clinical assessment, ultrasound examination, and urine culture
Atar[16]	2012	Spinal anesthesia in 18; general anesthesia in 1	19 (100%)	9.5F semi-rigid URS in 19 (holmium laser lithotripsy in 15 and stone forceps in 2)	Not mentioned	All 19 delivered at term	Dysuria-pain in 4; urinary infection in 1	Clavien-Dindo I in 4; Clavien-Dindo II in 1	Clinical assessment, US examination, and urine sample collection for culture and antibiogram
Bozkurt[17]	2012	Spinal anaesthesia in 22; general anaesthesia in 7; local anaesthesia in3	32 (100%)	9.5F semi-rigid URS (balloon dilator with pneumatic lithotripsy in 8, holmium laser in 17, then extracted with forceps; 2 extracted with forceps only)	100	All 32 delivered at term	Urinary infection in 4; dysuria-pain in 2; sepsis in 1; ureteral laceration in 2	Clavien-Dindo I in 4; Clavien-Dindo II in 4; Clavien-Dindo IV in 1	Obstetric care; clinical assessment, US examination, and urine samples for culture and antibiogram
Hoscan[18]	2012	Genaral anesthesia	57 (100%)	9.5F semi-rigid URS	85.3	Not mentioned	Urinary tract infection in 3; bladder irritation in 3; uterine contraction in 1	Clavien-Dindo I in 3; Clavien-Dindo II in 4	Obstetric care; clinical assessment, ultrasound examination, and urine culture
Johnson[19]	2012	General anesthesia in 32; local anesthesia in 5; epidural or spinal anesthesia in 9	46 (100%), 39 with stone	Flexible scope in 8, rigid scope in 21, Both scope in 17; Lithotripsy in 24, basket extraction in 37	86	44 delivered at term; preterm labor in 2	Not mentioned	Not mentioned	Not mentioned
Abdel[20]	2013	Spinal anesthesia	17 (100%), 13 with pneumatic lithoclast,4 with dormia extraction	7.3/8 F semi-rigid URS (Storz) and 6/7.5 F semi-rigid ureteroscope (Wolf)	100	All 17 delivered at term	0	0	Clinical assessment, abdominal ultrasonography, and urine culture and sensitivity. Radiographic imaging with KUB was done in the postpartum period
Bozkurt[21]	2013	Spinal anesthesia in 34; general anesthesia in 3; other in 4	41, 37 success (90.2%)	9.5F semi-rigid URS (laser lithotripsy in 27, pneumatic lithotripsy in 6 and stone extraction in 4)	85.5	All 41 delivered at term	Laceration in 3; perforation in 1; urinary infection in 4; dysuria-pain in 6; sepsis in 1	Clavien-Dindo I in 9; Clavien-Dindo II in 4; Clavien-Dindo III in 1; Clavien-Dindo IV in 1	Clinical assessment, US and urine samples for culture and antibiogram
Song[22]	2013	Epidural anesthesia in 21	21, 18 success (85.7%)	Wolf URS and LithoClastMaster	85.7	All 21 delivered at term	Hematuria in 2; stent-induced bladder irritation in 1	Clavien-Dindo I in 3;	Not mentioned
Keshvari[23]	2013	General anesthesia	44 (100%)	8F semi-rigid URS (pneumatic lithotripsy in 34, stone extraction with grasper in 16)	100	All 44 delivered at term	0	0	Obstetric care; clinical assessment, ultrasound examination, urinalysis and urine culture
Adanur[25]	2014	General anaesthesia without using halothane and nitric oxide	19 (100%)	7.5 F or 9.5 F semi-rigid URS (holmium-YAG laser in 19, a forcep for extraction of stone fragment in 9)	Not mentioned	All 19 delivered at term	Preterm urterin conrtaction in 1 and treated with tocolysi; urinary tract ingection in 1 and treated with appropriate antibiotics	Clavien-Dindo II in 2	Not mentioned
Georgescu[26]	2014	Spinal anesthesiain 42; general anesthesia 12	54, 44 success (81.5%)	Semi-rigid URS used during first 2 trimesters (32 success from 38 patients); flexible URS (12 from 16 cases) in the last trimester	Not mentioned	All 54 delivered at term; uterine contraction in 1	Urinary tract infection developed in 4 patients; renal colic in 2; prolonged hematuria in 1; stent-induced bladder irritation in 4	Clavien-Dindo I in 6; Clavien-Dindo II in 6	Obstetric care, clinical assessment, ultrasound examination, urinalysis and urine culture
Teleb[27]	2014	Spinal anaesthesia in 19; topical lidocaine anaesthesia with sedo-analgesia in 2	21 (100%)	9.5F semi-rigid URS (dilatation of ureteric orifice in 4, pneumatic lithoclast in 14, directly extracted stone in 7)	100	All 21 delivered at term	Urinary tract infection in 2; irritative bladder symptom in 4	Clavien-Dindo I in 4; Clavien-Dindo II in 2	US and urinalysis every 4 wks
Wang[28]	2014	Local anesthesia	64 (100%)	8/ 9.8F rigid URS (lithotripsy with Holmium:YAG laser)	81.3	All 64 delivered at term	Threatened abortion in 1; mild ureteric laceration in 1; mild bleeding in 5	Clavien-Dindo I in 6; Clavien-Dindo II in 1	Obstetric care; clinical assessment, ultrasound examination and urine culture
Fathelbab[29]	2016	Epidural anesthesia	41 (100%)	Semi-rigid URS ( pneumatic lithoclast in 22, directly extracted stone in 4)	89.7	All 41 delivered at term	Stent-related mild dysuria in 12; hematuria in 5,	Clavien-Dindo I in 17	Not mentioned
Zhang[30]	2016	General anesthesia in 72; spinal anesthesia in 45	117 (100%)	9.5F semi-rigid URS or flexible URS (pneumatic ballistic lithotripsy or Holmium:YAG laser)	84.6	All 117 delivered at term	Urosepsis in 1; threatened abortion in 12	Clavien-Dindo II in 12; Clavien-Dindo IV in 1	Obstetric care; clinical assessment, ultrasound examination, urinalysis and urine culture
Abedi[31]	2017	Not mentioned	45 (100%)	9.5F semi-rigid URS (holmium-YAG laser)	93.3	All 45 delivered at term	Preterm urterin conrtaction in 2 and treated with tocolysi; urinary tract ingection in 2 and treated with appropriate antibiotics	Clavien-Dindo II in 4	Not mentioned
Tan[32]	2018	General anesthesia or epidual anesthesia	23, 20 success (87%)	URS lithotripsy with pneumatic lithotripsy	Not mentioned	Not mentioned	Bladder irritation in 1; sliht hematuria in 1	Clavien-Dindo I in 2	Not mentioned

*URS* ureteroscopy; *SFR* stone-free rate

Meta-analysis indicated that there was no evidence of statistical heterogeneity between the two treatments with regards to operation success rate (Fig. 2, I^2^ = 12.1%, *P* = 0.280), normal fertility outcome (Fig. 3, I^2^ = 0.0%, *P* = 0.989) and adverse pregnant outcome (Fig. 4, I^2^ = 0.0%, *P* = 1.000). However, overall, complications for internal ureteral stent therapy were more common than for URS (Fig. 5, I^2^ = 91.0%, *P* < 0.001). We also analyzed pooled ORs for serious complications in the two treatments (Fig. 6). There was no evidence of significant statistical heterogeneity among the included studies (*I*^2^ = 0.0%, *P* = 1.000).

### Qualitative assessment and publication bias

The NOS tool was used to perform qualitative assessment of the selected studies to review the quality of the studies and detect possible bias (Tables 4 and 5). Of the 25 studies, eight were at a low risk of bias (7–9 stars); 16 studies were at a medium risk (4–6 stars), mainly due to bias from the representativeness of cases or controls, control definition and comparability. One study was at high risk (3 stars) mainly due to bad representativeness, lack of control and unclear control exposure. A funnel plot showed publication bias in the studies included in the meta-analysis (Begg's test with *P* < 0.001) (Additional file 1: Figure S1).

**Table 4:** Newcastle–Ottawa Scale review for cohort studies from systematic review

Study	Country	Selection				Comparability		Outcome			Total
		S1	S2	S3	S4	C1	C2	O1	O2	O3	
Liu et al. [14]	China	★	★	★	★			★	★	★	7
Bozkurt et al.[17]	Turkey	★	★	★	★			★	★	★	7
Teleb et al.[27]	Egypt	★	★	★	★			★	★	★	7

Guidelines for review

*Selection*

S1, Representativeness of the exposed cohort; ★a) representative of the community (e.g. community-based colorectal cancer-screening programme or registry) or (single hospital or clinic); b) selected group of people (e.g. nurses, volunteers); d) no description of the derivation of the cohort

S2, Selection of the non-exposed cohort: ★a) drawn from the same community as the exposed cohort; b) drawn from a different source; c) no description of the derivation of the non-exposed cohort

S3, Ascertainment of exposure: ★ a) secure record (eg medical records); ★b) structured interview; c) written self-report; d) no description

S4, Demonstration that outcome of interest was not present at start of study: ★ a)yes; b) no

*Comparability*

C1, ★ Study controls for one most important factor;

C2, ★ Study controls for any additional factors (1 > additional factors)

*Outcome*

O1, Assessment of outcome: ★a) independent blind assessment; ★b) record linkage; c) self-report; d) no description

O2, Follow-up was long enough for outcomes to occur (after delivery or longer): ★a) yes; b) no

O3, Adequacy of follow-up of cohorts: a) complete follow up—all subjects accounted for; b) subjects lost to follow up unlikely to introduce bias—small number lost > 10%; c) follow up rate < 90% and no description of those lost; d) no statement

**Table 5:** Newcastle–Ottawa Scale review for case–control and cross-sectional studies from systematic review

Study	Country	Selection				Comparability		Exposure			Total
		S1	S2	S3	S4	C1	C2	E1	E2	E3	
Ulvik et al.[8]	Norway	★	★					★		★	4
Scarpa et al.[9]	Italy	★	★					★		★	4
Parulkar et al. [10]	America	★	★	★	★			★	★	★	7
Lemos et al. [11]	Brazil	★						★		★	3
Rana et al. [12]	Pakistan	★	★					★		★	4
Elgamasy et al. [13]	Egypt	★	★					★		★	4
Polat et al. [15]	Turkey	★	★					★		★	4
Atar et al. [16]	Turkey	★	★					★		★	4
Bozkurt et al. [17]	Turkey	★	★					★		★	4
Hoscan et al. [18]	Turkey	★	★					★		★	4
Johnson et al. [19]	America	★	★					★		★	4
Abdel et al.[20]	Egypt	★	★					★		★	4
Song et al.[22]	China	★	★	★	★			★	★	★	7
Keshvari et al.[23]	Iran	★	★					★		★	4
Ngai et al. [24]	Iraq	★	★					★		★	4
Adanur et al. [25]	Turkey	★	★					★		★	4
Georgescu et al.[26]	Romania	★	★					★		★	4
Wang et al. [28]	China	★	★	★	★			★	★	★	7
Fathelbab et al. [29]	Egypt	★	★					★		★	4
Zhang et al. [30]	China	★	★	★	★			★	★	★	7
Abedi et al. [31]	Iran	★	★					★		★	4
Tan et al.[32]	China	★	★	★	★			★	★	★	7

Guidelines for review

*Selection*

S1, Case definition adequacy: ★a) requires independent validation (> 1 person/record/time/process to extract information, or reference to primary record source such as colonoscopy or medical/hospital records); b) record linkage or self-report with no reference to primary record; c) no description

S2, Representativeness of the cases: ★a) consecutive or obviously representative series of cases; b) potential for selection biases or not stated

S3, Selection of controls: ★a) community controls; b) hospital controls, within same community as cases; c) no description

S4, Definition of controls: ★a) no history of colorectal cancer or adenoma; b) no description of source

*Comparability*

C1, ★ Study controls for one most important factor;

C2, ★ Study controls for any additional factors (1 > additional factors)

*Exposure*

E1, Ascertainment of exposure: ★a) secure record (e.g. medical records); ★b) structured interview where blind to case/control status; c) interview not blinded to case/control status; d) written self-report or medical record only; e) no description

E2, Same method of ascertainment for cases and controls: ★a) yes; b) no

E3, Non-response rate: ★a) same rate for both groups; b) non respondents described; c) rate different and no designation

## Discussion

From the best of our knowledge, this is the first systematic review to investigate and compare the outcomes of ureteroscopy and serial D-J stenting therapy for pregnant females with urolithiasis. To determine the efficacy and safety of the two treatments, we analysed the available information in as much detail as possible. This meta‑analysis featured 25 studies with a total of 920 cases of urolithiasis during pregnancy. This meta‑analysis contained studies selected from several countries; as shown in Table 1, most studies originated from Asia (15 studies), followed by Africa (four studies), Europe (three studies) and America (including North and South America; three studies). Thus, this review represents a population of different ethnicities. Our analysis showed that operative success rates were almost the same for internal ureteral stents and URS (97% *vs.* 99%, *P* = 0.280). Internal ureteral stents were associated with more complications than URS (45% *vs.* 1%, *P* < 0.001); however, most complications were minor or could be adequately managed (serious complication rates were < 1% in the two groups, *P* = 1.000) and there was no statistical difference in normal delivery rate between the two treatments (99% *vs.* 99%, *P* = 0.989). In summary, both ureteroscopy and internal ureteral stents are safe and effective for pregnancy with symptomatic urolithiasis.

Urolithiasis in pregnancy is the most common non-obstetric reason for hospital admission; 80–90% of such cases are diagnosed in the 2^nd^ or 3^rd^ trimester of their pregnancy when the disease becomes symptomatic [33–36]. As the majority of calculi can be passed following the administration of intravenous fluids and analgesia, the first-line treatment for urolithiasis in pregnancy is conservative management. This is recommended by the latest guidelines from both the European Association of Urology (EAU) and the American Urological Association (AUA). However, if complications develop and affect fetal safety, or the patient does not experience adequate symptom relief, more aggressive treatments should be considered. Shock wave lithotripsy is absolutely contraindicated in pregnancy because of potential fetal death [37]. Percutaneous nephrostomy (PCN) drainage is also not an appropriate choice as it raises the risk of septic complications and imposes the additional burden of an external drain [38]. The common utilization of the prone position and fluoroscopy also represent limitations for the use of PCN in pregnancy [39]. Therefore, internal ureteral stents and URS are the most common treatments in the clinic for pregnant patients.

Following the failure of initial conservative treatment, the insertion of a D-J stent might be a safe choice. Serial stenting for pregnancy with urolithiasis is commonly used in clinic although there are not many relevant studies. After scanning articles over the past 30 years, only six related articles were included in this meta-analysis [10, 22, 24, 27, 28, 32]. Historically, serial stenting was considered as the gold standard of surgical treatment for pregnancy with urolithiasis as it was less invasive and could be performed under local anaesthesia [40]. This amount of anaesthetic and the reduced level of surgical trauma is considered to be safer for the fetus [24]. Our meta-analysis also indicated that this treatment relieves obstruction and pain while maintaining the pregnancy. However, there are still some negative opinions. On the one hand, serial stenting may be poorly tolerated by some pregnant women as it can cause pain and reduce the quality of life. On the other hand, insertion of a D-J stent is a temporary measure; such stents require regular replacement. Furthermore, the increased concentration of calcium and urate in urine during pregnancy can led to a tendency for encrustation; thus, these invasive operations need to be performed more frequently [20, 41]. However, an increase frequency of such invasive operations also leads to an increase in complications, including UTI and stent migration [27, 32, 42]; there is also an increase in cost [39]. Our meta-analysis demonstrated that the pooled ORs of complications after serial stenting was 45%. However, the pooled ORs for serious complications (Clavien-Dindo III-V) after serial stenting was < 1%. There was no evidence that serial stenting treatment was harmful for pregnancy as the pooled ORs for adverse pregnant outcomes was < 1%. Internal ureteral stents were thus proven to be safe for both the pregnant woman and the fetus.

Unlike internal ureteral stent operations, the use of URS to treat urolithiasis in pregnancy has been studied by many urologists; 23 papers were included in this meta-analysis [8, 9, 11–23, 25–32]. We found that the most common forms of anaesthesia were general and spinal. Although there are risks associated with anaesthesia and surgery, technological advancement provided a safeguard for perioperative safety. After systematic analysis, we calculated that the pooled ORs for complications was approximately 1% and the pooled ORs for normal fertility outcomes were 99%. Another advantage of URS was the high SFR (91%). High stone clearance rates and low complication rates made URS the recommended method in the 2020 EAU guideline. We noticed that most of cases of ureteroscopy involved the rigid option rather than the flexible option and that the choice of ureteroscope was related to the location of the stone. As shown in Table 1, most patients had stones located in the distal ureter; therefore, the rigid or semi-rigid ureteroscope was a more suitable choice.

In the latest 2020 EAU guidelines [6], URS appears to be the better selection for pregnancy with urolithiasis in comparison with internal ureteral stents while stent insertion therapy is only mentioned for symptomatic moderate-to-severe hydronephrosis during pregnancy. It appears that ureteral stent insertion is not an appropriate treatment for pregnant women with urolithiasis. However, the success of URS surgery depends on detailed preoperative preparation and stringent obstetric care. During emergencies or where there is a lack of obstetric care, an internal ureteral stent might be the better choice as it is also safe and effective and could gain time for URS later. Moreover, for pregnant females who do not want to take general anesthesia before childbirth, the insertion of a ureteral stent seems to be the only choice for relieving symptomatic urolithiasis. Urologists and obstetricians should work together to ensure the safety of the mother and fetus in such cases.

There were several inherent limitations to this meta‑analysis. First, most of the included studies were retrospective studies. This might cause inevitable methodological defects, including data bias, insufficient baseline comparison, and insufficient data collection. Urolithiasis during pregnancy is not a rare disease, but for urologists, it is not easy to handle both urolithiasis and obstetric care. After failed initial conservative treatment, such cases may become a urological emergency that requires a rapid response. Thus, well-designed RCTs are difficult to accomplish. Secondly, performance bias should also be considered. Although various centres perform similar operations, the medical equipment and medical teams are different. Surgery is a complex process; these differences may also lead to different outcomes. Furthermore, there was inevitable bias when the data were pooled. Therefore, further well-designed, prospective studies are required; these studies should take into account selection bias, performance bias and the issue of confounding. Finally, funnel plots showed certain publication bias in the included articles; however, we retained all of the studies as the sample size was small. Despite these limitations, this updated meta‑analysis provides an important clinical reference for urolithiasis during pregnancy.

## Conclusion

Although internal ureteral stents may cause minor complications, both ureteroscopy and internal ureteral stents showed less adverse effects on fertility results in pregnant women with symptomatic urolithiasis. Evidence suggests that URS therapy may have a greater advantage for pregnant women with urinary stones when the condition permits. As it has been proven to be safe and effective, internal ureteral stents can be considered in emergency or other special situations.

## Supplementary Information

**Additional file 1.** Search information, complication details, and result of publication bias.

## Data Availability

All data generated or analysed during this study are included in this published article and its supplementary information files.
