# Supplementary material for: Outcomes of ureteroscopy and internal ureteral stent for pregnancy with urolithiasis: a systematic review and meta-analysis
Source: BMC Urol. 2022 Sep 14;22:150. doi: 10.1186/s12894-022-01100-w (PMC9476568; doi:10.1186/s12894-022-01100-w)
Supplement: Supplementary file 1 — Additional file 1. Search information, complication details, and result of publication bias. [file 12894_2022_1100_MOESM1_ESM.docx]

Table S1. Search strategy and results.

|  | **Medline Search** | **Results** |
| --- | --- | --- |
| 1 | (Pregnanc* or Pregnancy or Pregnant or Gestation* or Pregnant woman or Mother*).mp. [mp=title, abstract, original title, name of substance word, subject heading word, floating sub-heading word, keyword heading word, organism supplementary concept word, protocol supplementary concept word, rare disease supplementary concept word, unique identifier, synonyms] | 1364603 |
| 2 | (Urinary Calcul* or Urinary Calculi or Urinary Calculus or Urinary Stone* or Urinary Tract Stone* or Ureteral Calcul* or Ureteral Calculi or Ureteral Calculus or Kidney Calcul* or Kidney Calculi or Kidney Calculus or Nephrolith or Renal Calcul* or Renal Calculi or Renal Calculus or Kidney Stone* or Staghorn Calcul* or Staghorn Calculi or Staghorn Calculus or Urinary Lithiasis).mp. [mp=title, abstract, original title, name of substance word, subject heading word, floating sub-heading word, keyword heading word, organism supplementary concept word, protocol supplementary concept word, rare disease supplementary concept word, unique identifier, synonyms] | 117989 |
| 3 | (Ureteroscopies or Ureteroscopic or Ureteroscopic Surgical or Ureteroscopic Surgical Procedure* or Ureteroscopic Surgery or Ureteroscopy).mp. [mp=title, abstract, original title, name of substance word, subject heading word, floating sub-heading word, keyword heading word, organism supplementary concept word, protocol supplementary concept word, rare disease supplementary concept word, unique identifier, synonyms] | 7810 |
| 4 | (Double-J stent or Ureteral stent or Ureteral double-J stent or Ureteral D-J stent or Double J ureteral stent or D-J ureteral stent or stent or D-J stent).mp. [mp=title, abstract, original title, name of substance word, subject heading word, floating sub-heading word, keyword heading word, organism supplementary concept word, protocol supplementary concept word, rare disease supplementary concept word, unique identifier, synonyms] | 100917 |
| 5 | 1 and 2 | 3673 |
| 6 | 3 or 4 | 107346 |
| 7 | 5 and 6 | 144 |
| 8 | limit 7 to yr="1980 -Current" | 144 |

|  | **Embase Search** | **Results** |
| --- | --- | --- |
| 1 | (Pregnanc* or Pregnancy or Pregnant or Gestation* or Pregnant woman or Mother*).mp. [mp=title, abstract, original title, name of substance word, subject heading word, floating sub-heading word, keyword heading word, organism supplementary concept word, protocol supplementary concept word, rare disease supplementary concept word, unique identifier, synonyms] | 1783332 |
| 2 | (Urinary Calcul* or Urinary Calculi or Urinary Calculus or Urinary Stone* or Urinary Tract Stone* or Ureteral Calcul* or Ureteral Calculi or Ureteral Calculus or Kidney Calcul* or Kidney Calculi or Kidney Calculus or Nephrolith or Renal Calcul* or Renal Calculi or Renal Calculus or Kidney Stone* or Staghorn Calcul* or Staghorn Calculi or Staghorn Calculus or Urinary Lithiasis).mp. [mp=title, abstract, original title, name of substance word, subject heading word, floating sub-heading word, keyword heading word, organism supplementary concept word, protocol supplementary concept word, rare disease supplementary concept word, unique identifier, synonyms] | 31030 |
| 3 | (Ureteroscopies or Ureteroscopic or Ureteroscopic Surgical or Ureteroscopic Surgical Procedure* or Ureteroscopic Surgery or Ureteroscopy).mp. [mp=title, abstract, original title, name of substance word, subject heading word, floating sub-heading word, keyword heading word, organism supplementary concept word, protocol supplementary concept word, rare disease supplementary concept word, unique identifier, synonyms] | 12638 |
| 4 | (Double-J stent or Ureteral stent or Ureteral double-J stent or Ureteral D-J stent or Double J ureteral stent or D-J ureteral stent or stent or D-J stent).mp. [mp=title, abstract, original title, name of substance word, subject heading word, floating sub-heading word, keyword heading word, organism supplementary concept word, protocol supplementary concept word, rare disease supplementary concept word, unique identifier, synonyms] | 224637 |
| 5 | 1 and 2 | 625 |
| 6 | 3 or 4 | 233357 |
| 7 | 5 and 6 | 161 |
| 8 | limit 7 to yr="1980 -Current" | 161 |

|  | **Web of science** | **Results** |
| --- | --- | --- |
| 1 | TS=(Pregnanc* or Pregnancy or Pregnant or Gestation* or Pregnant woman or Mother*) | 84124 |
| 2 | TS=(Urinary Calcul* or Urinary Calculi or Urinary Calculus or Urinary Stone* or Urinary Tract Stone* or Ureteral Calcul* or Ureteral Calculi or Ureteral Calculus or Kidney Calcul* or Kidney Calculi or Kidney Calculus or Nephrolith or Renal Calcul* or Renal Calculi or Renal Calculus or Kidney Stone* or Staghorn Calcul* or Staghorn Calculi or Staghorn Calculus or Urinary Lithiasis) | 12638 |
| 3 | TS=(Ureteroscopies or Ureteroscopic or Ureteroscopic Surgical or Ureteroscopic Surgical Procedure* or Ureteroscopic Surgery or Ureteroscopy) | 793 |
| 4 | TS=(Double-J stent or Ureteral stent or Ureteral double-J stent or Ureteral D-J stent or Double J ureteral stent or D-J ureteral stent or stent or D-J stent) | 13690 |
| 5 | #1 AND #2 | 1862 |
| 6 | #3 OR #4 | 14521 |
| 7 | #5 AND #6 | 153 |

|  | **Cochrane library** | **Results** |
| --- | --- | --- |
| 1 | Pregnanc* or Pregnancy or Pregnant or Gestation* or Pregnant woman or Mother* | 97230 |
| 2 | Urinary Calcul* or Urinary Calculi or Urinary Calculus or Urinary Stone* or Urinary Tract Stone* or Ureteral Calcul* or Ureteral Calculi or Ureteral Calculus or Kidney Calcul* or Kidney Calculi or Kidney Calculus or Nephrolith or Renal Calcul* or Renal Calculi or Renal Calculus or Kidney Stone* or Staghorn Calcul* or Staghorn Calculi or Staghorn Calculus or Urinary Lithiasis | 14165 |
| 3 | Ureteroscopies or Ureteroscopic or Ureteroscopic Surgical or Ureteroscopic Surgical Procedure* or Ureteroscopic Surgery or Ureteroscopy | 973 |
| 4 | Double-J stent or Ureteral stent or Ureteral double-J stent or Ureteral D-J stent or Double J ureteral stent or D-J ureteral stent or stent or D-J stent | 15134 |
| 5 | #1 AND #2 | 1852 |
| 6 | #3 OR #4 | 15698 |
| 7 | #5 AND #6 | 61 |
| 8 | Restrict to trials | 41 |

| Table S2. Complications and their Clavien-Dindo Classification | |  |
| --- | --- | --- |
| **Clavien-Dindo I** | | |
| Postoperative fever without antibiotics | | |
| Passed D-J stent | | |
| Irritative bladder symptom | | |
| Ureteral laceration | | |
| Dysuria-pain | | |
| Uterine contraction without drug treatment | | |
| Stent drop | | |
| Hematuria | | |
| **Clavien-Dindo II** | |  |
| Postoperative fever/urinary tract infection with antibiotics treatment |  |  |
| Stent replaced |  |  |
| Uterine contraction with drug treatment |  |  |
| **Clavien-Dindo III** | |  |
| D-J stent migration requiring operation |  |  |
| Stent symptoms requiring early removal |  |  |
| Calcified stent requiring operation |  |  |
| Ureteral perforation or avulsion |  |  |
| **Clavien-Dindo IV** | |  |
| Preterm labor or abortion |  |  |
| Urosepsis |  |  |
| **Clavien-Dindo V** | |  |
| Death |  |  |
